# Nogo-B protects mice against lipopolysaccharide-induced acute lung injury

**DOI:** 10.1038/srep12061

**Published:** 2015-07-15

**Authors:** Wujian Xu, Ying Zhu, Yunye Ning, Yuchao Dong, Haidong Huang, Wei Zhang, Qinying Sun, Qiang Li

**Affiliations:** 1 Department of Respiratory Disease, Changhai Hospital, Second Military Medical University, China; 2 Department of Respiratory Medicine, Jinling Hospital, Nanjing, China; 3 Department of Respiratory Medicine, Shanghai First People’s Hospital, Shanghai Jiaotong University School of Medicine, China

## Abstract

Nogo-B, a member of the reticulon 4 protein family, plays a critical role in tissue repair and acute inflammation. Its role in acute lung injury (ALI) remains unclear. Here, we assessed the function of Nogo-B during tissue injury in a lipopolysaccharide (LPS)-induced ALI mouse model. We found that pulmonary Nogo-B was significantly repressed after LPS instillation in C57BL/6 mice. Over-expression of pulmonary Nogo-B using an adenovirus vector carrying the Nogo-B-RFP-3flag gene (Ad-Nogo-B) significantly prolonged the survival of mice challenged with a lethal dose of LPS. The Ad-Nogo-B-treated mice also had less severe lung injury, less alveolar protein exudation, and a higher number of macrophages but less neutrophil infiltration compared with Ad-RFP-treated mice. Interestingly, microarray analysis showed that the Ad-Nogo-B-treated mice had different gene expression profiles compared with the controls and the prominent expression of genes related to wound healing and the humoral immune response after LPS induction. Of the 49 differently expressed genes, we found that the expression of PTX3 was significantly up-regulated following Nogo-B over-expression as observed in lung tissues and RAW264.7 cells. In conclusion, Nogo-B plays a protective role against LPS-induced ALI, and this effect might be exerted through the modulation of alveolar macrophage recruitment and PTX3 production.

Acute lung injury and acute respiratory distress syndrome (ALI/ARDS) are serious clinical disorders characterized by the disruption of the capillary-alveolar barrier, leading to the development of pulmonary edema and acute respiratory failure^1^. Although multiple new treatment strategies have been introduced, the incidence and mortality of this disease remain high in patients^2^.

Bacterial infection is a leading cause of ALI/ARDS. The development of infection induced ALI is a multi-step process involving a variety of inflammatory cells and mediators. Small numbers of microbes can be eliminated by resident defenses such as alveolar macrophages, whereas larger numbers of virulent microbes require the activation of innate immunity, mainly macrophages and neutrophils. The immune system normally maintains a fine balance between defense against microorganism invasion and the prevention of tissue injury^3^. During the development of ALI/ARDS, this balance is disrupted. A chain of inflammatory cells and mediators is over-activated, and the mechanisms for resolving inflammation are also impaired, ultimately resulting in persistent lung injury^4^,^5^. Further investigation into how this process is regulated will increase the understanding of the pathophysiology of ALI/ARDS and provide insights into potential treatments.

Reticulon 4 (Rtn4), also called neurite outgrowth inhibitor (Nogo), is a protein with three main isoforms Nogo-A, B and C^6^,^7^. Nogo-A was originally identified in the central nervous system as a potent inhibitor of axonal growth and repair^8^. Nogo-B, which is localized to the endoplasmic reticulum and cellular membrane, is abundant in peripheral tissues including lung tissues^9^,^10^. It plays pivotal roles in vascular repair and regeneration^9^,^11^, hepatic fibrosis^12^, asthma^13^, cancer development^14^,^15^ and key steps in inflammation including macrophage recruitment^11^ and leukocyte transmigration^16^. Nogo-B-deficient mice show reduced vessel regeneration after vascular ischemia injury^11^ and reduced hepatocyte proliferation and liver regeneration after liver injury^17^. Its deficiency also results in increased apoptosis in hepatic stellate cells^12^, suggesting a beneficial role in hepatic cirrhosis. Furthermore, mice lacking Nogo-B exhibit decreased macrophage infiltration in injured tissues^11^ and reduced neutrophil recruitment to sites of inflammation^16^, indicating that it plays an important role in modulating macrophage and neutrophil recruitment under inflammatory conditions. Given the key roles of macrophages and neutrophils and their related inflammatory mediators in ALI/ARDS, the role of endogenous Nogo-B in the development of pulmonary inflammation and injury during ALI deserves further investigation.

We report herein that pulmonary Nogo-B expression was significantly reduced in the lungs of a LPS-induced ALI mouse model. The over-expression of Nogo-B in the lungs significantly prolonged survival and attenuated the severity of lung injury in ALI mice. This protein may exert its effects by modulating the recruitment of inflammatory cells and the secretion of inflammatory mediators and by promoting PTX3 expression.

## Methods

### Animal models of ALI

Six- to eight-week-old male C57BL/6 mice (Shanghai Laboratory Animal Company, China) were anesthetized with 5% chloral hydrate intraperitoneally. To induce ALI, the mice were intra-tracheally instilled with 15 mg/kg or 25 mg/kg of LPS (*E. coli* O111:B4, Sigma, U.S.A) and then sacrificed for assessment at the indicated times^18^. All experimental protocols involving mice were approved by the Second Military Medical University animal care and use committee. The animals were treated humanely according to the institutional animal care guidelines.

### Adenovirus

Adenoviral vectors carrying mouse Nogo-B with a red fluorescence protein-3flag (RFP-3flag) tag (Ad-Nogo-B) or RFP-3flag (Ad-RFP) gene alone were constructed by SBO Medical Biotechnology Co., Ltd. (Shanghai, China)^19^. Seven days before LPS instillation, a dose (1 × 10^9^ pfu) of adenovirus was administered to the mice via intra-tracheal instillation^20^. For cell transfection, RAW264.7 cells was seeded at a density of 2 × 10^5^ cells per well for a 24-well culture plate and transfected with the adenovirus (MOI = 200). The efficacy of the fusion proteins was evaluated by western blotting and immunofluorescence staining.

### Histology

Mouse lungs were embedded in paraffin and processed for histological analysis as previously described^21^. Lung sections were stained with hematoxylin and eosin (HE) and subjected to lung injury assessment. Nogo-B expression was examined by immunohistochemistry using a rabbit anti-mouse Nogo-B antibody (1:100 dilution; Abcam, UK). For each section, five randomly chosen fields were used to determine the lung injury score according to the existence of alveolar edema/exudates, hemorrhage, and interstitial/alveolar cellular infiltration with scores ranging from 1 to 3 (0, absent; 1, mild; 2, moderate; and 3, severe)^22^. For frozen sections, fresh lung tissues were embedded in Optimal Cutting Temperature Compound (SAKURA, U.S.A) and snap frozen at –80°C. Then, 6-μm-thick frozen sections were produced using a cryostat (Leica, German) and fixed in acetone for 3 minutes.

### Bronchial alveolar lavage fluid (BALF) analysis

BAL was performed as previously reported^23^. Briefly, a 1.0-ml aliquot of PBS was instilled into the lungs of the mice through the trachea and then carefully removed three times. BAL cells were collected (2000 rpm, 10 min), stained with Diff-quick stain solution (Baso, China) and subjected to a blinded manual cell count. The supernatant was collected for measurements of the BAL protein and cytokine concentration (Bradford assay; Bio-Rad, CA).

### Cytokines measurement^24^

The levels of tumor necrosis factor-α (TNF-α) and macrophage chemotaxis protein-1 (MCP-1) in the BALF were determined by ELISA (eBioscience, U.S.A) following standard protocols.

### Western blotting^25^

Lung homogenates (right lower lobe) were dissolved and boiled in Laemmli buffer for 5 min. Twenty micrograms of protein was subjected to electrophoresis on 12% SDS-PAGE gels and transferred to nitrocellulose membranes. The membranes were blocked in PBS containing 5% skimmed milk for 2 h at room temperature and then reacted with a primary antibody against Nogo-B or 3flag. The quantity of the sample was normalized based on the level of GAPDH (Abcam, UK).

### Immunofluorescence staining^26^

For immunofluorescence staining, frozen lung sections were incubated with an anti-Nogo-B antibody overnight at 4°C. 4¢ 6-Diamidino-2-phenylindole (DAPI) was used for nuclear staining.

### Microarray analysis^27^

For microarray analysis, four array groups were prepared (i.e., mice treated with or without LPS after Ad-Nogo-B or Ad-RFP transfection) and were tested with two biological replicates for each group. Microarray hybridization and data analysis were performed by Phalanx Biotech Group (Phalanx Biotech Group, Taiwan) and re-analyzed using ArrayTrack software^28^. Briefly, total RNA from lung homogenates was extracted using TRIzol (Invitrogen, U.S.A.), and RNA integrity was determined by Agilent RNA 6000 Nano assay. Targets were prepared using an Eberwine-based amplification method with Amino Allyl MessageAmp II aRNA Amplification Kit (Ambion, AM1753) to generate amino-allyl antisense RNA. Aminoallyl-RNA probes labeled with NGS-Cy5 were hybridized at 50°C for 16 h to a Mice Whole Genome OneArray^®^ Version 2.0. The hybridized array was scanned with an Axon 4000B Scanner and analyzed with Rosetta Resolver System^®^. Differentially expressed genes were those with a log2 (fold change) > = 1.0 and a *P *< 0.05. The detailed experimental protocols and array data are available at http://www.ncbi.nlm.nih.gov/geo/query/acc.cgi?acc = GSE48787 under the accession number GSE48787.

### Cell culture and siRNA transfection^10^

Murine leukemia virus transformed RAW264.7 cells were grown in DMEM containing 10% fetal bovine serum (Gibco, U.S.A). Cells were cultured in a standard humidified incubator at 37 °C with 5% CO_2_. For 6-well plate transfection, RAW264.7 cells were transfected with 300 ng siRNA using 12 μl HiPerFect (Qiagen, Germany) in 100 μl Opti-M culture medium (Gibco, U.S.A). All-Star Negative Control siRNAs (NEGi forward: UUCUCCGAACGUGUCACGU, reverse: ACGUGACACGUUCGGAGAA) and Nogo-B-specific siRNAs (NOGOi forward: GGAUCUCAUUGUAGUCAUATT, reverse: UAUGACUACAAUGAGAUCCAT) were purchased from Qiagen. The efficacy of siRNA interference was assessed at 60 h for mRNA expression and at 72 h for protein expression using real-time PCR and western blotting, respectively.

### Real-time PCR^10^

Total RNA was extracted from lung tissues and cells with TRIzol Reagent (Invitrogen, U.S.A). Real-time PCR was performed using ABI 7500 and a SYBR Prime Script^TM^ RT Reagent Kit (TaKaRa, Japan) following the manufacturers’ instructions. The primers used for quantitative real-time PCR are listed in table 1. The results were normalized to the level of β-actin mRNA in each sample.

### Statistical analysis

All values are expressed as the mean ± standard deviation of n observations (n ≧ 3). Statistical analyses were performed using one-way analysis of variance (ANOVA) for multiple comparisons. Student’s *t* test was used to assess differences between the two groups. Log-rank tests were performed using Kaplan-Meier survival curves. A two-tailed *P* value of less than 0.05 was considered to be statistically significant.

## Results

### Reduction of the pulmonary Nogo-B level in LPS-induced ALI mice

**Figure 1:**
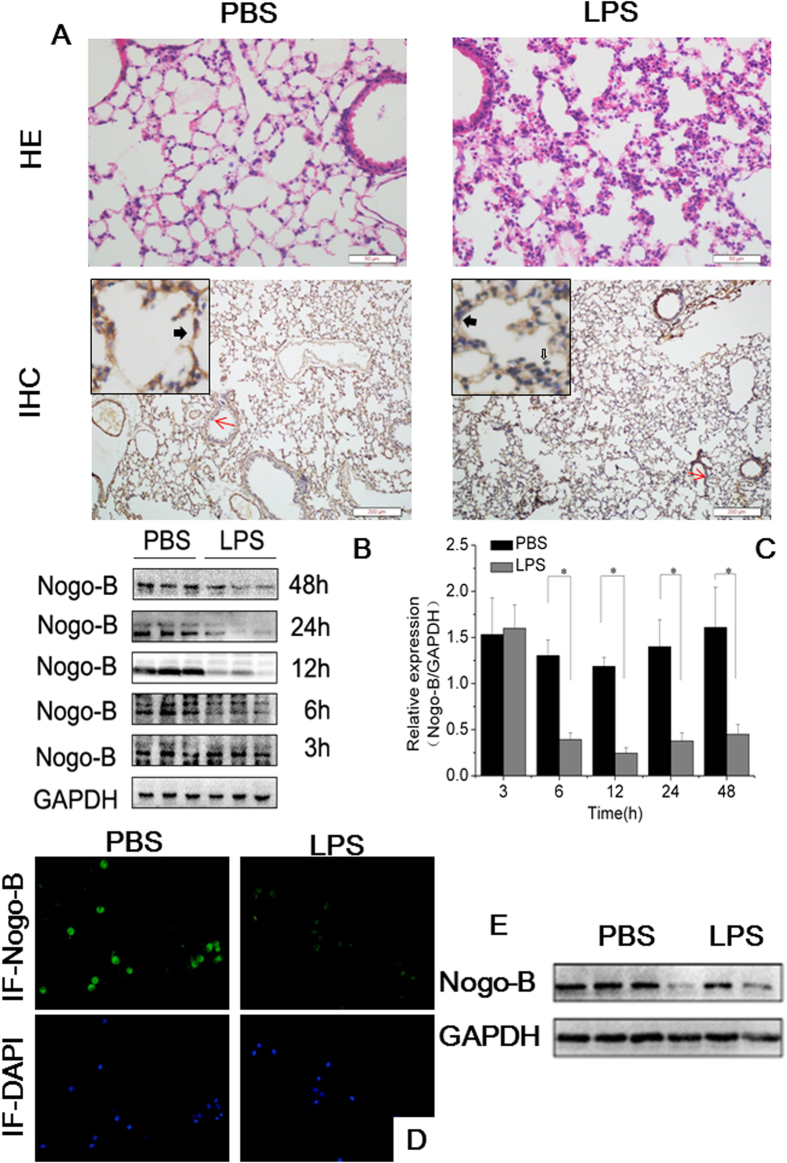
The down-regulation of Nogo-B in the lungs and BALF cells of mice with ALI. Naïve C57BL/6 mice were challenged with LPS by intra-tracheal instillation or with PBS and sacrificed 12 h later (n = 5 for each group). (**A**) Representative images of HE stained tissues showing the extent of lung injury (upper lane) and lung tissues immunostained for Nogo-B (down lane). The solid black arrowheads indicate Nogo-B expression in alveolar epithelial cells. The hollow arrowheads indicate Nogo-B expression in inflammatory cells. The red arrows show Nogo-B expression in airway epithelium. (**B**) Representative western blotting analysis of Nogo-B in lung homogenates at the indicated time points show a reduction in its level after LPS instillation. GAPDH was used as an internal control (anti-Nogo-B concentration 1:3000; for full blots, see Supplemental Materials S1). (**C**) Band intensity analysis of the western blotting results revealed the significant down-regulation of Nogo-B at the indicated time points. n = 5 for each group. The data are presented as the mean ± standard deviation (SD). *P < 0.05. Expression of Nogo-B in BALF cells was also detected at 12 h by immunofluorescence staining (**D**) and western blotting, n = 3 for each group. (**E**).

To investigate the role of Nogo-B in ALI, we initially exposed naïve C57BL/6 mice to LPS (15 mg/kg) by intra-tracheal instillation. At 12 h after LPS challenge, histological examination showed typical lung injury, including alveolar congestion, hemorrhage, inflammatory cells infiltrations and thickness of the alveolar wall (Fig. 1A). The IHC results demonstrated that Nogo-B was normally highly expressed in bronchial epithelial cells, vascular endothelial cells and alveolar epithelial cells in naïve mice. At 12 h after LPS induction, Nogo-B expression in bronchial epithelial and vascular endothelial cells was slightly down-regulated, whereas its expression in the alveolar epithelium was greatly repressed (Fig. 1A).

To further reveal the role of Nogo-B in the development of ALI, mouse lung tissues were collected at different time points after LPS challenge (3–48 h) and were analyzed for Nogo-B expression. Western blotting showed that pulmonary Nogo-B expression was significantly reduced at 6 h after LPS instillation and exhibited a 4.8-fold down-regulation compared with the level in controls at 12 h. After that time point, Nogo-B expression slowly recovered at 24 h and 48 h, but the levels were still significantly lower than those in the controls (Fig. 1B–C, Figure S1A). In addition, this reduction in Nogo-B expression in response to LPS instillation at 12 h was also found in BALF cells by immunofluorescence staining and Western blotting (Fig. 1D–E, Figure S1B). These results strongly suggest that Nogo-B participates in the development of LPS-induced ALI.

### Nogo-B over-expression prolonged the survival time of mice with ALI induced by a fatal dose of LPS

**Figure 2:**
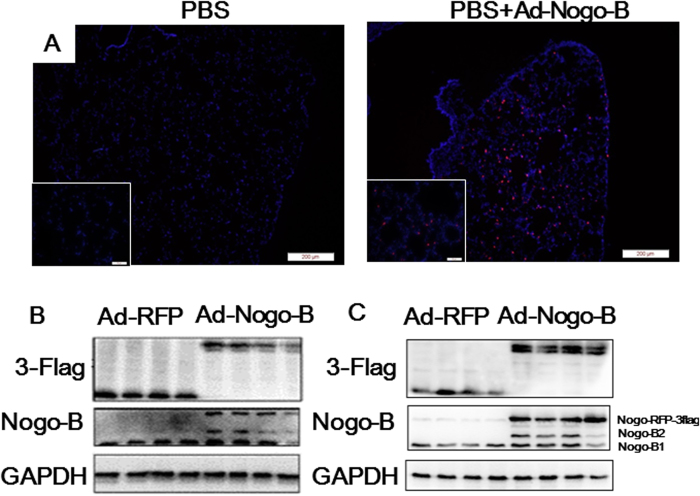
Over-expression of Nogo-B in the lungs and BALF cells. Naïve C57BL/6 mice were intra-tracheally instilled with Ad-RFP or Ad-Nogo-B adenovirus. (**A**) Representative slides of frozen lung sections at 7 days after adenovirus instillation. The Nogo-B-RFP-3flag fusion protein is represented by red fluorescence and is localized to the cytoplasm. Nuclei are indicated by blue fluorescence. Western blot analysis of the Nogo-B fusion protein using an anti-Nogo-B or anti-3-flag antibody showed the over-expression of Nogo-B in the lung tissues (**B**) and BALF cells (**C**). n = 4 for each group.

To determine the biological function of Nogo-B repression in ALI, adenovirus-mediated Nogo-B expression was used to prevent the physiological down-regulation of this protein after LPS stimulation. An adenovirus vector expressing an RFP-3flag-tagged form of Nogo-B (Ad-Nogo-B) was constructed and transfected into naïve C57BL/6 mice to induce the over-expression of pulmonary Nogo-B through tracheal instillation. None of the mice that received either type of adenovirus transfection died during the experiment. In the frozen lung tissue sections, the Nogo-B fusion protein was identified in the alveolar wall, confirming the over-expression of Nogo-B in lung tissues (Fig. 2A). Western blotting analysis revealed that the Nogo-B-RFP-3flag fusion protein was present in both the lung tissues and BALF cells at 7 days after adenovirus transfection (Fig. 2B–C), suggesting that the instillation of adenovirus can effectively up-regulate pulmonary Nogo-B levels.

**Figure 3:**
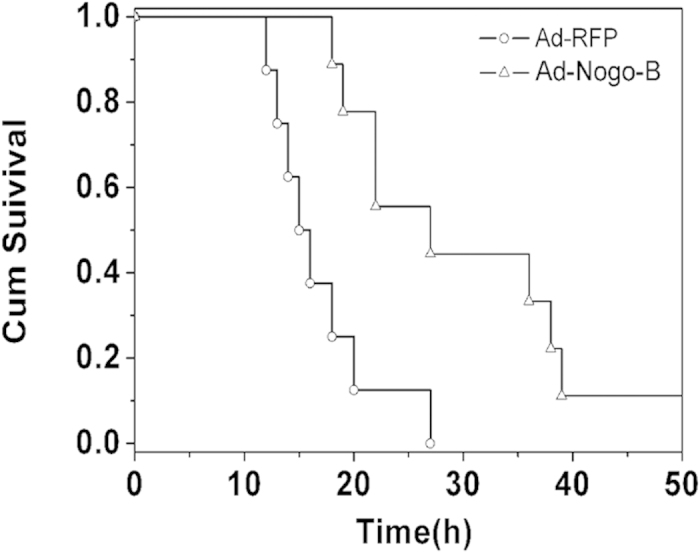
Nogo-B over-expression prolonged the survival time of mice with LPS-induced ALI. Mice over-expressing Ad-RFP or Ad-Nogo-B were challenged with a lethal dose of LPS (25 mg/kg). The survival time was recorded over 48 h. n = 8 for each group.

Next, a lethal dose of LPS (25 mg/kg) was intra-tracheally instilled into mice over-expressing Nogo-B-RFP or RFP, and their survival times were recorded. The Ad-RFP-treated mice all died by 27 h, whereas one mouse survived to 48 h in the Ad-Nogo-B-treated group (Fig. 3). The survival time was significantly longer for the Ad-Nogo-B-treated mice than for the Ad-RFP-treated mice (28.7 ± 10.78 *vs.* 16.9 ± 4.85 h, n = 8, *P *< 0.05).

### Nogo-B over-expression ameliorated lung injury induced by LPS in mice

**Figure 4:**
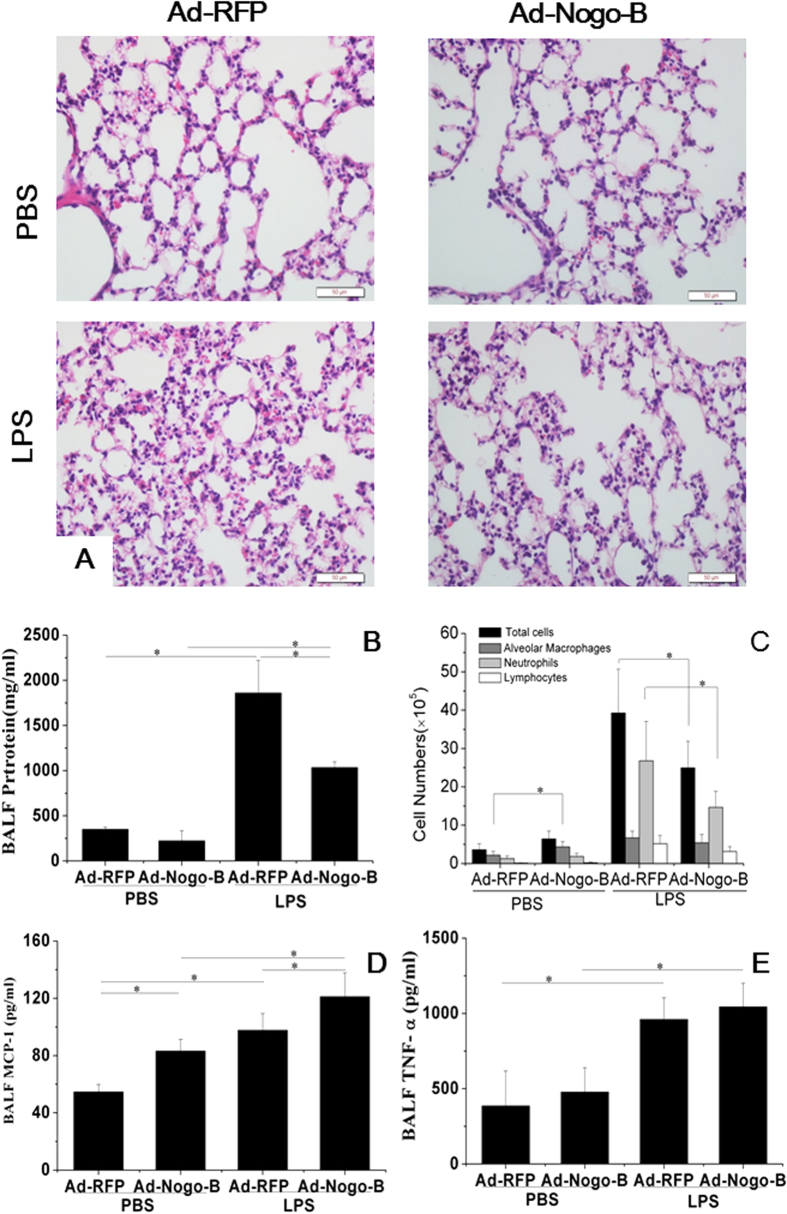
Nogo-B over-expression ameliorated LPS-induced acute lung injury. (**A**) Representative images of HE-stained tissues showing the extent of lung injury in Ad-RFP- and Ad-Nogo-B-treated mice before and after LPS instillation. (**B**) BALF protein levels were determined by Bradford protein analysis. (**C**) Cell counts in the BALF from Ad-RFP- or Ad-Nogo-B-treated mice before and after LPS instillation. The MCP-1 (**D**) and TNF-α (**E**) levels in the BALF of Ad-RFP- and Ad-Nogo-B-treated mice were determined by ELISA. n = 5 for each group. The data are the mean ± SD, *P < 0.05 as indicated.

To assess the effect of Nogo-B on LPS-induced lung injury, mice transfected with Ad-Nogo-B or Ad-RFP were challenged with LPS by intra-tracheal instillation (15 mg/kg) for 12 hours. LPS-induced lung injury was observed in the Ad-RFP- and Ad-Nogo-B-treated mice, manifesting as hemorrhage, the infiltration of inflammatory cells into alveoli and lung parenchyma, and alveolar wall thickening. The lung injury score was lower in the Ad-Nogo-B-treated mice compared with the Ad-RFP-treated mice (3.2 ± 0.1 *vs.* 4.3 ± 0.2, *P *< 0.01) (Fig. 4A). The BALF total protein concentration in the Ad-Nogo-B group was significantly lower than that in the Ad-RFP group (1032.9 ± 67.47 *vs.* 1859.2 ± 359.76 μg/ml, n = 5, *P *< 0.05), indicating less protein leakage in the Ad-Nogo-B-treated mice (Fig. 4B).

To assess the pulmonary inflammatory response, BALF cells and cytokines were evaluated (Fig. 4C). Before LPS induction, the Ad-Nogo-B-treated mice showed higher macrophages numbers in the BALF than the Ad-RFP-treated mice. At 12 h after LPS instillation (15 mg/kg), the total numbers of BALF cells and neutrophils were lower in the Ad-Nogo-B-treated mice than in the Ad-RFP-treated mice. These results showed that Nogo-B over-expression promoted macrophage infiltration but inhibited neutrophil recruitment. The Ad-Nogo-B-treated mice also had higher MCP-1 levels both before and after LPS instillation, but there was no significant difference in the TNF-α concentration between the two groups (Fig. 4D–E).

### Nogo-B affected the expression of PTX3 after LPS induction

**Figure 5:**
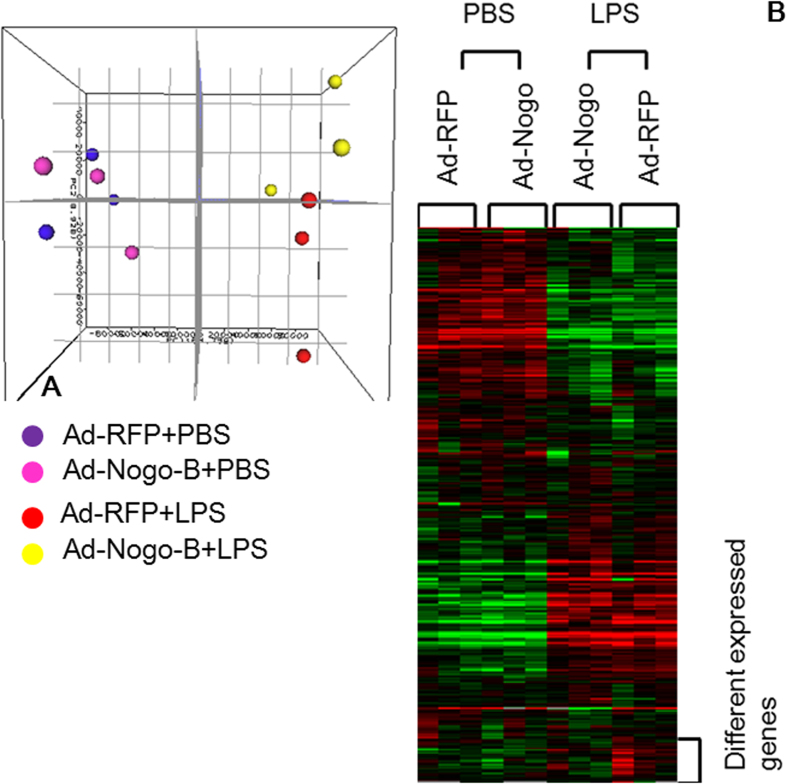
Effects of Nogo-B over-expression on gene expression profiles after LPS instillation in C57BL/6 mice. (**A**) Principal component analysis showed that the gene expression profiles were separated based on Ad-Nogo-B transfection after LPS induction. (**B**) Hierarchical clustering analysis demonstrated that the gene expression patterns were highly dependent on LPS induction and were affected by Nogo-B over-expression.

To further determine the mechanisms by which Nogo-B is modulated in LPS-induced lung injury, microarray was performed to detect differentially expressed genes between the mice transfected with either Ad-Nogo-B or Ad-RFP and subsequently treated with or without LPS (15 mg/kg). PCA and clustering analysis showed that the overall gene profiles were separated based on Ad-Nogo-B transfection after LPS induction but not before LPS induction (Fig. 5A-B). After LPS induction, the Ad-RFP-treated mice showed a total of 1,988 differentially expressed genes. Of these genes, gene ontology analysis revealed those assigned to the biological process subontology were mainly involved in locomotory behavior and the inflammatory response (Table 2).The Ad-Nogo-B-treated mice showed 1,645 differentially expressed genes after LPS instillation. Gene ontology analysis revealed that these genes were involved in locomotory behavior and the inflammatory response, in addition to the response to wounding, response to external stimulus and humoral immune response (Table 2), suggesting that Nogo-B over-expression exerts a broader effect on the pathophysiology of LPS-induced ALI.

**Figure 6:**
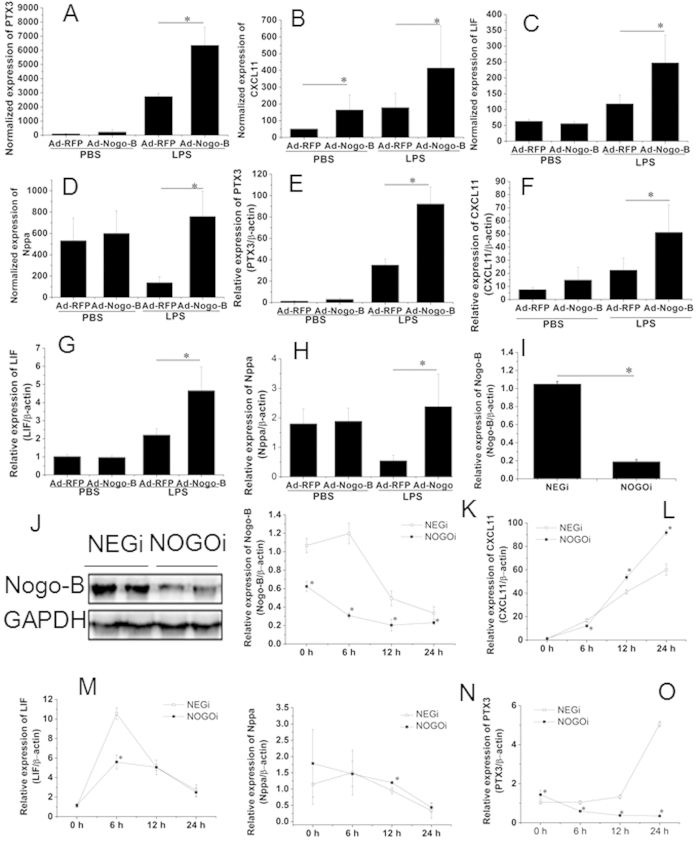
Down-regulation of Nogo-B inhibited PTX3 expression in RAW 264.7 cells. The expression levels of PTX3, LIF and CXCL11 in Ad-Nogo-B transfected mice were compared with those in Ad-RFP transfected mice treated with or without LPS. The microarray results are shown in A-D. The real-time PCR results normalized to the level of β-actin are listed in E-H. n = 6 for each group. Mean ± SD. *P < 0.05 as indicated. Nogo-B specific siRNA significantly inhibited the mRNA (I) and protein (G) levels of Nogo-B after siRNA transfection. The cells were then treated with LPS (1 μg/ml) for 24 h, and Nogo-B (K), LIF (L), CXCL11 (M), Nppa (N), and PTX3 (O) mRNA levels were detected using real-time PCR at 0 h, 6 h, 12 h and 24 h. n = 3 for each group. Mean ± SD. *P < 0.05 as indicated.

A total of 43 up-regulated and 6 down-regulated genes were detected in the Ad-Nogo-B treated mice compared with the Ad-RFP mice after LPS induction. The top 20 differentially expressed genes are listed in Table 3. The number of differentially expressed genes between these two groups was not sufficient to conduct GO and pathway analyses; therefore, we performed a search to evaluate the relationships of these genes with ALI using PubMatrix (http://pubmatrix.grc.nia.nih.gov) and conducted a manual search in PubMed. Of these genes, long pentraxin 3 (PTX3), chemokine (C-X-C motif) ligand 11 (CXCL11), leukemia inhibitor factor (LIF), and natriuretic peptide type A (Nppa) have been reported to play critical roles in modulating neutrophil recruitment and tissue injury in LPS-induced ALI. Real-time PCR analysis confirmed that at 12 h after LPS instillation, the PTX3, LIF, CXCL11, and Nppa levels in the Ad-Nogo-B-treated mice were 2.62, 2.53, 2.0 and 4.35 -fold higher than those in the Ad-RFP-treated mice, respectively (Fig. 6A–H).

**Figure 7:**
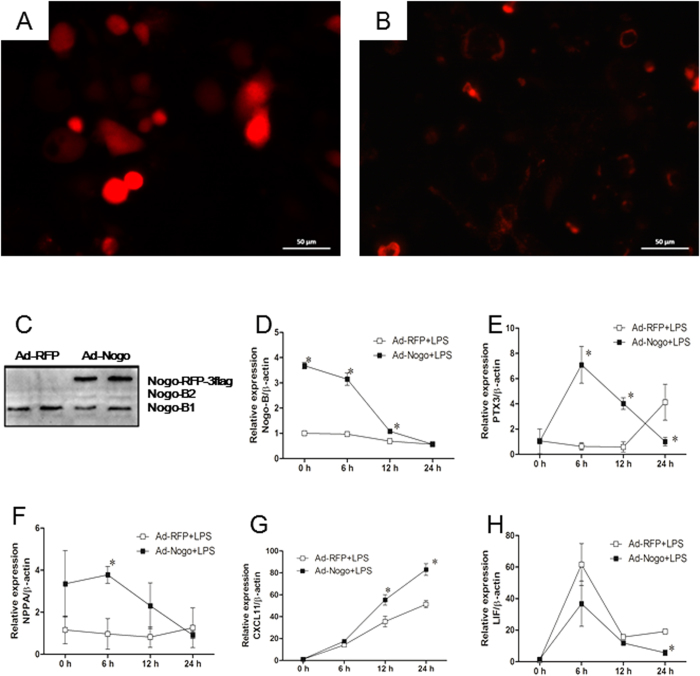
Nogo-B over-expression promoted PTX3 expression in RAW264.7 cells. Raw264.7 cells transfected with Ad-RFP (**A**) or Ad-Nogo-B (**B**) showed RFP expression under fluorescence microscopy. Western blotting revealed the Nogo-B-RFP-flag protein in Ad-Nogo-B treated cells (**C**). D-H, time course of mRNA expression of Nogo-B (**D**), PTX3 (**E**), Nppa (**F**), CXCL11 (**G**), and LIF (**H**) over the 24 hours LPS incubation. n = 3 for each group. Mean ± SD. *P < 0.05 as indicated.

To further verify these results, RAW264.7 cells were transfected with Nogo-B specific siRNA (NOGOi) or Nogo-B adenovirus vectors to manipulate Nogo-B expression. siRNA interference resulted in the significant down-regulation of Nogo-B mRNA and protein levels (Fig. 6G–H). The cells were then incubated with LPS (1 μg/ml) for 24 h, resulting in the further down-regulation of Nogo-B mRNA in NOGOi groups compared with the NEGi groups. The CXCL11 (**J**), LIF (**K**) and Nppa (**L)** mRNA levels were markedly elevated as early as 6 h, but only slight differences (<2-fold) were detected between the Nogo-B-down-regulated groups and controls during the 24 h challenge. The PTX3 mRNA level in the control cells showed a slow but significant elevation during the LPS challenge, whereas in the NOGOi-treated cells, its level was markedly down-regulated and was 14.3-fold lower than that of the controls at 24 h (**L**). We further assessed the candidate genes by conducting an over-expression experiment. The over-expression of Nogo-B in RAW 264.7 cells (Fig. 7A–D) resulted in significantly increases in the PTX3 at 6 h and 12 h compared with those in the controls (Fig. 7E). The Nppa and CXCL11 expression levels were also slightly elevated after LPS incubation (Fig. 7F–G). No significant differences in expression were observed for LIF following LPS challenge (Fig. 7H). Thus, only PTX3 expression showed a significant change in relation to Nogo-B expression, suggesting it may act downstream of Nogo-B.

## Discussion

In the present study, we aimed to determine whether and how Nogo-B might affect ALI/ARDS. We first found that Nogo-B expression in the lungs was greatly suppressed in mice with LPS-induced ALI, suggesting that it is involved in the development of acute inflammation and the related lung injury. Nogo-B is highly expressed in the lungs, kidneys, liver and cardiovascular systems. Previous studies have revealed that endogenous Nogo-B plays roles in vessel regeneration^29^, tissue repair^17^, inflammation^16^ and apoptosis^12^,^30^. Clinical evidence indicates that in patients with asthma^13^, atherosclerosis^31^ and aneurysms^32^, local Nogo-B expression is inhibited. In accordance with these results, in animal models of acute or chronic asthma^10^,^13^ and vascular injury^9^, Nogo-B has also been found to be down-regulated. However, two groups have recently reported that Nogo-B levels are greatly increased during vessel ischemia^11^ and liver cirrhosis^33^, suggesting that it has complex roles under different pathological conditions.

The over-expression of pulmonary Nogo-B prolonged the survival time of mice, alleviated lung injury and reduced alveolar protein exudation and neutrophil infiltration. In addition, it increased MCP-1 secretion and the number of alveolar macrophages (AMs) in the alveolar space, in accordance with a previous study showing that Nogo-B knockout mice exhibit impaired MCP-1 and CCR2 induction upon tissue injury^11^. These results suggest that Nogo-B has a protective role against ALI.

Balance is key to the development of ALI/ARD. Accumulating evidence indicates that AMs play a complex role in the development of this condition. Although AMs are the primary cells that recognize LPS, recruit neutrophils and initiate the cascade of inflammatory reactions^34^, sufficient AMs are also essential for the inhibition of excessive neutrophil accumulation and inflammatory responses. Beck *et al.* have reported that the depletion of AMs leads to a significant increase in leukocyte infiltration and the aggravation of lung injury^35^. In addition, Maus *et al.* have reported that blocking MCP-1 using a polyclonal antibody results in higher levels of capillary-alveolar barrier damage and myeloperoxidase in ALI rats^36^. Furthermore, CC chemokine receptor 2 (CCR2)-knockout mice show the reduced recruitment of AMs and, greater neutrophil infiltration and severity of lung injury^37^. Because MCP-1 is a potent cytokine involved in macrophage migration and recruitment, these results suggest that sufficient MCP-1 secretion and AM recruitment are also protective factors in ALI. Therefore, the increase in the numbers of AMs induced by Nogo-B expression before LPS stimulation might be a reason for the reduced neutrophil recruitment in mice with LPS-induced ALI. Another explanation may be the elevated MCP-1 levels. In addition to being a chemotactic factor for macrophages, MCP-1 itself functions as a protective factor in ALI by promoting N_2_O production through the MCP-1/CCR2 pathway, as reported by Okuma *et al.*^38^. However, we did not find any difference in TNF-α secretion following LPS challenge. TNF-α is an early phase cytokine that reaches its peak level as early as 4 h after induction, indicating that Nogo-B may not modulate early inflammatory responses.

A previous study has shown that only one gene is differentially expressed between Nogo-B-deficient and wild-type mice at baseline^13^. In accordance with this finding, our PCA results showed that the gene profiles of the Ad-Nogo-B- and Ad-RFP-treated mice only differed after LPS induction, suggesting that Nogo-B may mainly function under stress conditions. Using microarray and PubMatrix analysis, we identified 4 genes (LIF, Nppa, CXCL11, and PTX3) that might function downstream of Nogo-B over-expression. LIF is known to inhibit leukocyte migration in LPS-induced lung inflammation, and its elevation in ALI is beneficial for the alleviation of inflammation^39^. CXCL11 is a cytokine for activated T cells^40^, which binds CXCR3 with high affinity and has the potential to restrain inflammatory autoimmunity^41^. Interestingly, Nppa, a peptide mainly secreted by atrial cells that plays a key role in relieving heart preload, was also among the differentially expressed genes. Its expression has been recently found to be elevated in ARDS, and administration of exogenous Nppa has been shown to successfully attenuate both gram- negative and positive bacterial infection-induced ALI^42^,^43^. Although the microarray results suggested that these genes might be involved in Nogo-B-related protection in ALI, *in vitro* studies did not show clear trends among the expression of the three above mentioned genes and the Nogo-B level.

Only PTX3 was regulated by both Nogo-B knockdown and overexpression in the LPS-induced ALI models. PTX3 is an acute-phase protein that is produced by a variety of tissue cells at the site of infection or inflammation^44^. Previous studies have shown that it is up-regulated in LPS- or high-volume ventilation-induced ALI murine models^45^. Although a high PTX3 level is accompanied by severe ALI/ARDS^46^, Han *et al.*^22^ have recently reported that PTX3-knockout ALI mice show elevated neutrophil infiltration and cell death and more severe lung injury after LPS induction, suggesting a protective role of PTX3 in ALI. In addition, PTX3 can bind P-selectin and attenuate neutrophil recruitment at sites of inflammation^47^. Therefore, higher levels of PTX3 secretion induced by the over-expression of Nogo-B might inhibit neutrophil infiltration, thus decreasing the severity of lung injury. However, the precise mechanisms by which PTX3 is regulated by Nogo-B require further investigation.

Unlike the results of a previous study, which has shown that mice lacking Nogo-B exhibit a marked reduction in neutrophil transmigration under ketamine-, xylazine- or carrageenan-induced acute inflammation^16^, we found that Nogo-B-over-expressed mice had less neutrophil infiltration upon LPS stimulation. This discrepancy might be due to differences in the properties of the stimuli and the disease pathophysiology. In addition, due to the limited efficacy of adenovirus transfection, the microarray only identified 49 differentially expressed genes in association with severe inflammation. Thus, further investigation of Nogo-B knockout mice is warranted.

In conclusion, Nogo-B may act as an upstream factor to modulate neutrophil infiltration by promoting alveolar macrophage recruitment and PTX3 expression, and it plays a protective role in LPS-induced ALI.

## Additional Information

**How to cite this article**: XU, W. *et al.* Nogo-B protects mice against lipopolysaccharide-induced acute lung injury. *Sci. Rep.* **5**, 12061; doi: 10.1038/srep12061 (2015).

## Supplementary Material

Supplementary Information

## Figures and Tables

**Table 1:** primer sequences for real-time PCR.

Targets	Forward primer (5ʹ – 3ʹ)	Reverse primer (5ʹ – 3ʹ)
PTX3	ACTTCATCCCACCGAGGACC	ATCATGCTGGAGAACTCGCAG
LIF	GGAGTCCAGCCCATAATGAA	TGAGCTGTGCCAGTTGATTC
CXCL11	TGAGATGAACAGGAAGGTCACA	AACTTTGTCGCAGCCGTTACTC
Nogo-B	GCAGGGGCTCGGGCTCAGTGG	GTTCACATGACCAAGAGCAG
Nppa	TGAAAAGCAAACTGAGGGCT	CAGAGTGGGAGAGGCAAGAC
β-actin	CCTGTACGCCAACACAGTGC	ATACTCCTGCTTGCTGATCC

**Table 2:** Top 5 bio functions of biological process of LPS induced genes affected by Ad-Nogo-B transfection, as analyzed by GO analysis.

Comparisons	Gene Name	Genes in geneset	Genes in overlap	p-value
Ad-RFP *vs*. Ad-RFP+LPS	Locomotory behavior	91	32	0.00616
	Inflammatory response	124	37	0.0509
	Negative regulation of phophorylation	12	5	0.122
	Response to wounding	185	50	0.123
	Response to external stimulus	306	78	0.181
Ad-Nogo-B *vs*. Ad-Nogo-B+LPS	Inflammatory response	124	37	0.00209
	Response to wounding	185	50	0.00401
	Locomotory behavior	91	28	0.00433
	Response to external Stimulus	306	75	0.00769
	Humoral immune response	31	11	0.0233

**Table 3:** Top 20 differentially expressed genes between Ad-Nogo-B and Ad-RFP treated mice after LPS instillation.

Gene Symbol	Gene name	Gene ID	Fold Change Ad-Nogo-B/d-RFPA	*p*-value
Inmt	indolethylamine N-methyltransferase	NM_009349.3	0.42	0.000003
Mob1a	MOB kinase activator 1A	NM_145571.2	0.44	0.01373
Fam101a	family with sequence similarity 101, member A	NM_028443.2	0.47	0.048129
Pnmal1	PNMA-like 1	NM_001007569.1	0.48	0.020917
Nppa	natriuretic peptide type A	NM_008725.2	5.44	3.26E-08
Kng2|Kng1	kininogen 2|kininogen 1	NM_001102409.1	4.20	2.29E-17
Cngb3	cyclic nucleotide gated channel beta 3	NM_013927.2	3.23	0.007987
Rspo3	R-spondin 3 homolog (Xenopus laevis)	NM_028351.3	2.90	0.028219
Ttc14	tetratricopeptide repeat domain 14	NM_027619.3	2.58	0.028555
Htra4	HtrA serine peptidase 4	NM_001081187.3	2.58	0.000015
Tpm1	tropomyosin 1, alpha	NM_001164254.1	2.57	0.019978
Cxcl11	chemokine (C-X-C motif) ligand 11	NM_019494.1	2.53	0.018486
Bpifa1	BPI fold containing family A, member 1	NM_011126.3	2.52	0.00035
B4galnt4	beta-1,4-N-acetyl-galactosaminyl transferase 4	NM_177897.3	2.42	0.000076
Myl7	myosin, light polypeptide 7, regulatory	NM_022879.2	2.42	0.045599
Myoz2	Myozenin2	NM_008987.3	2.38	3.42E-09
Ptx3	pentraxin related gene	NM_008987.3	2.37	3.42E-09
Ifit1	Interferon induced protein with tetratricopeptide repeats 1	NM_008331.3	2.36	0.000004
IL2ra	Interleukin 2 receptor, alpha chain	NM_008367.3	2.31	0.000615
LIF	Leukemia inhibitor factor	NM_001039537.1	2.01	0.001755
