# Supplementary material for: Nogo-B protects mice against lipopolysaccharide-induced acute lung injury
Source: Sci Rep. 2015 Jul 15;5:12061. doi: 10.1038/srep12061 (PMC4502524; doi:10.1038/srep12061)
Supplement: Supplementary Information [file srep12061-s1.doc]

**Title**： Nogo-B protects mice against lipopolysaccharide-induced acute lung injury

**Authors:**

Wujian XU^1, 2#^, Ying ZHU^1#^, Yunye Ning^1^, Yuchao DONG^1^, Haidong HUANG^1^, Wei ZHANG^1^, Qinying SUN^1^, Qiang LI^3^*

**Institutes**:

1. Department of Respiratory Disease, Changhai Hospital, Second Military Medical University, China

2. Department of Respiratory Medicine, Jinling Hospital, Nanjing University School of Medicine, Nanjing, China.

3. Department of Respiratory Medicine, Shanghai First People’s Hospital, Shanghai Jiaotong University School of Medicine, China.

# These authors contributed equally to this study.

***Corresponding author**:

Prof. LI Qiang

Department of Respiratory Diseases

Shanghai First People’s Hospital, Shanghai Jiaotong University School of Medicine,

No.600, Yishan Road,

Shanghai 200233, China.

**Tel.**: +86-13801602220

**Fax**: +86-21-65302653

**Email**: liqressh@hotmail.com


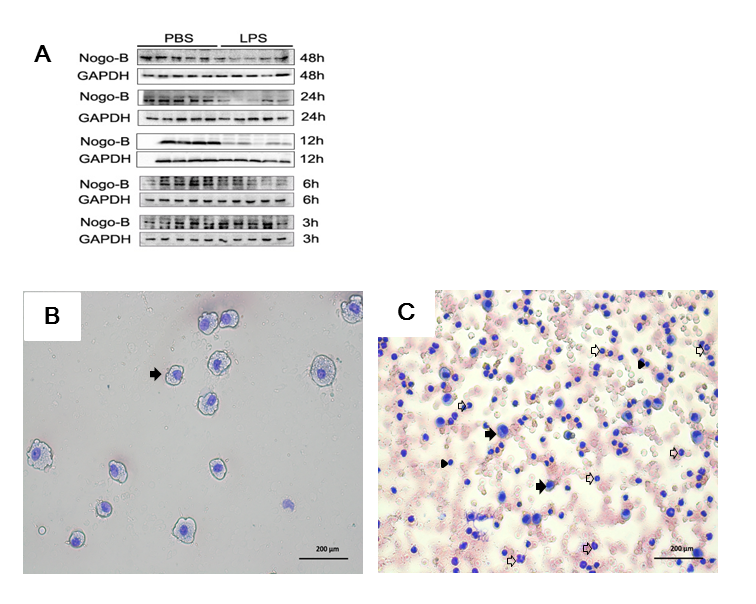
 **Figure S1: Pulmonary Nogo-B expression and BALF cell staining in PBS- or LPS-treated mice**. A, Nogo-B levels in lung homogenates were detected with rabbit-anti-mouse-Nogo-B antibody overnight at 4℃ (1:3000, Abcam) and then incubated with HRP conjugated anti-rabbit secondary antibody (1:2000, Jackson ) using western blotting. The blotted PVDF membrane was scanned in FluorChem FC3 (Alpha). Diff-quick staining of BALF cells from PBS (B) or LPS (C) treated mice. Black solid arrowheads indicate alveolar macrophages, hollow arrowheads indicate neutrophils, and black solid arrows indicate lymphocytes. Bar = 200 μm.
